# The medial septum-hippocampus-lateral septum circuitry in spatial memory: linking healthy function to early Alzheimer’s disease and translational opportunities

**DOI:** 10.1186/s40035-025-00511-7

**Published:** 2025-09-29

**Authors:** Yujie Song, Junjun Ni, Hong Qing, Zhenzhen Quan

**Affiliations:** 1 Key Laboratory of Molecular Medicine and Biotherapy, School of Life Science, Beijing Institute of Technology, Beijing, 100081 China; 2 Department of Biology, Shenzhen MSU-BIT University, Shenzhen, 518172 China

**Keywords:** Alzheimer's disease, Spatial memory, Medial septum–hippocampus–lateral septum neural circuitry, Synaptic dysfunction, Neuromodulation, Glutamate receptors

## Abstract

Hippocampus (HPC)-associated spatial memory deficits are one of the earliest symptoms of Alzheimer’s disease (AD). Current pharmacological treatments only alleviate the symptoms but do not prevent disease progression. The emergence of neuromodulation technology suggests that specific neural circuits are potential therapeutic targets for AD. Current studies have analyzed the medial septum (MS)–HPC and the HPC–lateral septum (LS) circuitries separately. A comprehensive understanding of their synergistic effects and overall dysregulation in AD remains limited. In this review, we will integrate anatomical and functional evidence to give an overview of the role of the MS–HPC–LS circuitry in spatial memory, the mechanisms of AD-related dysregulation, and therapeutic strategies targeting the circuitry, specially focusing on molecular interventions (receptor modulation) and bioengineering strategies (circuit-specific stimulation).

## Introduction

Alzheimer's disease (AD) is a progressive neurodegenerative disorder that currently affects more than 35 million people worldwide. With the increasing global life expectancy, the number of individuals with AD is estimated to reach around 150 million by 2050. Epidemiological data on AD suggest that the number of patients will continue to increase, placing a significant burden on healthcare systems [1]. However, there is still no effective treatment for AD, as clinical trials targeting molecular pathologies such as β-amyloid (Aβ) and tau pathology have failed. Most existing treatments can only provide temporary relief of symptoms, but cannot prevent worsening of the disease [2, 3]. Moreover, an increasing body of evidence suggests that AD is linked to the impairment of specific crucial neural circuits in the central nervous system (CNS) [4, 5]. The emergence of the neuromodulation technology suggests the potential of specific neural circuits as new therapeutic targets for AD [5]. Treatments targeting specific neural circuits may become the key to the treatment of AD-related spatial memory deficits.

Spatial memory dysfunction is an important feature of AD and often manifests at the earliest stages of the disease, before the onset of other cognitive impairments, such as language and working memory impairments [6–8]. Patients may be unable to accurately perceive their location and orientation, and have difficulty remembering routes and familiarizing themselves with the layout of their environment, affecting all aspects of their daily life. AD patients have deficits in both egocentric and allocentric spatial memory abilities [6, 9]. Serino et al*.* have shown that spatial impairment is a diagnostic indicator of AD cognitive decline [6, 8, 10, 11], and can be used to monitor disease progression or assess presymptomatic AD [12]. The dysfunction of spatial memory in the early stages of AD has become a focus of diagnosis and research. Notably, spatial memory is a core component of the declarative memory system in humans and shares key neuroanatomical substrates with episodic memory [13, 14]. In animal research, spatial navigation tasks such as the Morris water maze are commonly used to assess memory performance. In human studies, spatial disorientation correlates closely with early dysfunction of the hippocampus (HPC) and septal regions in AD [15, 16]. Therefore, spatial memory represents a quantifiable cognitive phenotype across species and serves as an important translational bridge linking mechanistic insights from animal models to early cognitive symptoms observed in clinics.

Normal cognitive functions of the brain rely on the coordination of multiple key neural circuits [17]. The HPC plays a central role in processing memory and cognition-related functions [18–20]. Accumulating studies have shown that the HPC is involved in the encoding, storage and retrieval of spatial information in the brain, forming tight neural circuits with other limbic system structures [21, 22]. The medial septum (MS)–HPC–lateral septum (LS) circuitry supports the learning and cognitive functions through complex bi-directional connections and information exchange [23, 24]. Abnormalities in the structure and function of this circuitry can lead to cognitive impairment [25, 26]. In individuals at early stages of AD, there is a reduction in the integrity of the HPC and its associated cortical networks [27]. This phenomenon has also been observed in mouse models, where degeneration of bidirectional anatomical connections between the MS and the HPC is evident in 4.5-month-old AD mice, and functional connectivity within the LS–HPC neural circuit is also disrupted in younger mice [25, 28]. This indicates that altered circuit connectivity may be an important neurobiological basis for their spatial memory dysfunction. In addition, synapses that function as circuit connections may be damaged [4]. Glutamate receptor-mediated synaptic dysfunction in AD is considered one of the key factors leading to cognitive decline [29]. In particular, abnormal function of glutamate receptors in the HPC and its associated neural circuits may impair synaptic plasticity, thereby affecting learning and memory abilities [30, 31]. Glutamate receptor-mediated synaptic dysfunction at the level of the MS–HPC–LS neural circuitry may be a key therapeutic target for the treatment of AD-related spatial memory impairment.

Although the MS–HPC and the HPC–LS pathways have been individually studied in relation to spatial memory and AD, systematic investigation of the MS–HPC–LS circuitry as a functional unit remains rare. On the one hand, the circuitry spans multiple brain regions with distinct structural and functional characteristics. Earlier experimental techniques lacked the capacity to simultaneously manipulate and monitor activity across such a distributed network, thus limiting in-depth exploration of its integrative function. On the other hand, although the LS is a major downstream of the HPC, it has traditionally been associated with emotional regulation and has long been overlooked in AD-related cognitive research [32]. In recent years, however, the role of the LS in spatial memory has received increasing attention. Development of advanced neurotechnologies such as multi-region optogenetic manipulation, fiber photometry, and miniature microscopes, has enabled integrated studies of cross-regional circuits and laid the technical foundation for revisiting the role of the MS–HPC–LS circuitry in cognitive function [33, 34]. Notably, recent findings also suggest that the LS sends feedback projections to the MS, indicating that the MS–HPC–LS circuitry may not only support unidirectional information flow but may also form a closed-loop structure capable of dynamic regulation [33]. This anatomical feature provides a potential mechanism for the coordinated interaction of these regions in spatial cognition and further underscores the need to study them as a unified functional circuitry.

In this review, we first overview the connectivity of the MS–HPC–LS circuitry under normal physiological conditions, as well as how its circuit dysfunction leads to spatial memory impairments in AD. We further discuss the mechanisms of MS–HPC–LS neural circuitry damage in AD spatial memory dysfunction in terms of synaptic deficits. Finally, we explore novel therapeutic approaches and bioengineering strategies that rescue the MS–HPC–LS neural circuitry in AD.

## Integrated structural–functional organization of the MS–HPC–LS circuitry in the normal brain

The MS, HPC, and LS each play distinct roles in spatial memory processing and are interconnected through topographically organized projections, forming a highly integrated neural circuit [35, 36]. As the principal subcortical driver of hippocampal theta rhythms (4–12 Hz rhythmic brain activity associated with learning, memory encoding, and movement coordination), the MS provides upstream rhythmic modulation critical for spatial information encoding [36]. The HPC encodes spatial locations and contextual cues via place cell activity, oscillatory coupling, and synaptic plasticity [37, 38]. The LS, a key subcortical output of the HPC, receives structured input and transforms spatial signals into motivational and behavioral responses [39]. These regions interact through well-defined feedforward and limited feedback pathways, forming a functionally coordinated network with partially closed-loop features. The integrity of this circuit may be essential for proper spatial memory encoding and retrieval. The MS–HPC–LS circuitry in rodents and humans shows topographical correspondence. While the MS and LS are anatomically well-defined in rodents, the human septum verum is thought to encompass homologous regions, though clear anatomical subdivisions remain undefined [40]. This supports its cautious inclusion in translational circuit-level studies. In this section, we will outline the structural and functional characteristics of MS and LS, and examines how their interactions with the HPC contribute to rhythmic coordination, spatial processing, and behavioral output.

### MS: a central hub for rhythmic output

#### Anatomical structure and neuronal subtype composition

The MS, together with the diagonal band of Broca (DBB), forms the MS-DBB complex, a core component of the MS–HPC–LS circuitry located in the dorsomedial part of the septal area (including MS and LS) [23, 41]. In some studies, the MS and DBB are considered as a single anatomical unit due to their close structural continuity. The MS is located beneath the corpus callosum, along the midline between the lateral ventricles, and lacks clear morphological boundaries or defined anatomical subdivisions. Neurons within the MS are organized in a concentric "onion-like" pattern, arranged from inner to outer layers based on their biochemical markers [42, 43].

The MS primarily comprises three types of neurons, including cholinergic (ChAT^+^) neurons, γ-aminobutyric acidergic (GABAergic) neurons, and glutamatergic (Glu^+^) neurons. The cholinergic neurons express choline acetyltransferase (ChAT) and are predominantly distributed in the outer layers. The GABAergic neurons are mainly located in the inner layers and also express glutamic acid decarboxylase (GAD) and markers such as parvalbumin (PV), calbindin, and nerve growth factor. Glutamatergic neurons, which release glutamate, include a subset that co-expresses ChAT or GAD, while another subset lacks both markers [44, 45]. These neuronal subtypes are interconnected locally, forming a dense microcircuit network that constitutes the architectural foundation for intra-septal information processing [46]. This organized cellular architecture provides the anatomical substrate for the role of MS as a pacemaker in hippocampal oscillatory coordination.

#### Rhythm modulation and downstream synchronization mechanisms

The MS acts as a rhythmic pacemaker within the limbic system, playing a dominant role in the generation and maintenance of theta oscillations. Its functional integrity is essential for the emergence of hippocampal theta rhythms and for synchronizing rhythmic activity across downstream limbic structures [47, 48]. As one of the most prominent subcortical inputs to the HPC, the MS regulates both the frequency and the synchrony of hippocampal oscillatory activity via its diverse efferent projections [49, 50].

The three principal neuronal subtypes in the MS contribute differentially to rhythm modulation. The GABAergic neurons, especially the PV⁺ population, constitute the core of the MS rhythmic output. GABAergic neurons serve as key temporal synchronizers. Their firing is tightly phase-locked to theta cycles. Rather than increasing the firing rate directly, these neurons enhance the rhythmic propensity and entrain downstream structures via hippocampal interneuronal networks [49–52]. The ChAT⁺ neurons do not exhibit theta phase-locked firing, instead, they play an essential role in shaping transitions between hippocampal network states. Activation of MS ChAT⁺ neurons increases the hippocampal theta amplitude while suppressing sharp-wave ripples (SWRs, brief high-frequency oscillatory events commonly considered biomarkers of memory consolidation), suggesting a state-gating function of ChAT⁺ neurons that modulates the selection of rhythmic modes [48, 53, 54]. The Glu⁺ neurons modulate local circuit coordination. When activated, these Glu⁺ neurons can induce hippocampal theta synchronization across multiple frequency bands, although their modulatory effects depend mainly on local septal circuits rather than direct projections via the fornix. Optogenetic activation of their axonal terminals alone does not alter hippocampal theta power, indicating that their primary function lies within local MS network coordination [55].

The regulation of rhythmic activity within the MS depends on the functional specialization and coordinated interaction of distinct neuronal subtypes [56]. The GABAergic, ChAT⁺, and Glu^+^ neurons contribute to phase synchronization, network state transitions, and local circuit integration, respectively, working together to sustain the stability and flexibility of theta oscillations across the MS–HPC system. Experimental studies have shown that lesions or functional inhibition of the MS significantly reduces the amplitude of HPC theta rhythms, further confirming its critical role in theta rhythmogenesis [57, 58].

Together, these distinct yet complementary neuronal functions enable the MS to regulate hippocampal oscillatory states essential for spatial memory processing.

#### MS neuronal subtypes exhibit stage-specific functions in spatial memory

Beyond their role in rhythmic modulation, the neuronal subtypes in MS exhibit clear functional specialization across different behavioral phases of spatial memory processing, including memory encoding, task execution, and retrieval stages.

During the memory encoding stage, the MS GABAergic neurons project to HPC interneurons in the dentate gyrus (DG), where they regulate the inhibitory tone onto granule cells. A reduction in MS-DG GABAergic input leads to disinhibition of granule cells, causing transient overexcitation, thereby destabilizing spatial memory encoding [59]. These findings indicate that MS plays a critical role in the initial filtering and gating of spatial information.

In the task execution phase, the firing rate of MS Glu^+^ neurons correlates positively with the locomotor speed, indicating their role in rhythm-movement coupling that stabilizes navigation and path continuity [60]. Meanwhile, cholinergic neurons in the MS are essential for the goal-directed behavior during task execution. Selective inhibition of the MS–HPC ChAT⁺ neurons impairs performance in target-location association tasks, and immunotoxin-induced ablation of ChAT⁺ neurons disrupts the object-place memory and context-goal tasks across multiple paradigms [61–64]. These results highlight the broad involvement of the cholinergic system in maintaining task strategies and behavioral execution.

Finally, in the retrieval and consolidation phase, the MS–HPC cholinergic transmission is crucial for memory consolidation. In sepsis models, the MS-HPC cholinergic signaling is impaired, accompanied by reduced hippocampal long-term potentiation (LTP), while activation of this pathway rescues both synaptic plasticity and cognitive performance [65]. Moreover, activation of cholinergic neurons during the delay phase of spontaneous alternation tasks suppresses SWRs and impairs strategic decision-making. In the goal-location tasks, such activation reduces SWR occurrence in target zones and compromises learning efficiency. During sleep, this cholinergic activation enhances theta-gamma coupling (gamma, 30–100 Hz fast oscillatory activity involved in attention, learning, and inter-regional coordination), suggesting a role in regulating the temporal precision of memory retrieval through rhythm-dependent mechanisms [61, 66].

### The MS–HPC circuit: rhythm generation and spatial memory regulation

#### Anatomical organization and bidirectional connectivity

The MS forms reciprocal connections with the HPC through the fimbria-fornix pathway, projecting primarily to the CA1 and CA3 subregions and targeting both ipsilateral and contralateral hippocampal areas. These anatomical links provide the structural basis for MS involvement in rhythm modulation and the bidirectional information exchange within the septohippocampal system [67].

The three principal neuronal types in the MS exhibit distinct projection patterns to the HPC. The GABAergic neurons primarily innervate hippocampal interneurons to modulate network oscillatory regulation. The cholinergic neurons, which account for approximately 60% of MS–HPC projections, predominantly target pyramidal cells and display widespread, unmyelinated axonal arborizations [68–71]. The Glu^+^ neurons, comprising ~ 23% of MS neurons, mainly project to CA1 interneurons and to a lesser extent to CA3 pyramidal neurons [72]. In the CA1 region, the terminal distribution of MS-derived inputs differs markedly among postsynaptic cell types: the interneurons receive approximately 67% GABAergic, 21% glutamatergic, and 12% cholinergic inputs, whereas the pyramidal neurons receive 27%, 7%, and 66%, respectively, highlighting a clear target-specific projection bias [72].

In the reverse direction, GABAergic interneurons in the HPC, predominantly somatostatin-positive (SST⁺) neurons, which comprise ~ 93% of the feedback population, project back to the MS and primarily innervate septal GABAergic and cholinergic neurons [73, 74]. Intriguingly, a subset of PV⁺ interneurons may participate in both ascending and descending pathways, potentially serving dual regulatory functions in septohippocampal coordination [75].

Together, the highly organized and reciprocal connectivity between the MS and the HPC is the anatomical foundation for their coordinated involvement in rhythm generation and spatial memory regulation, and underpins the functional integration of the MS–HPC–LS circuitry.

#### Rhythm generation and spatial memory regulation

The MS–HPC projections serve as a central rhythm-generating hub for spatial memory system. Within the MS–HPC circuit, GABAergic, cholinergic, and Glu^+^ neurons regulate hippocampal rhythmic activity through distinct projection pathways, collectively contributing to the encoding, consolidation, and retrieval of spatial memory [36].

GABAergic neurons enhance hippocampal theta and gamma synchronization primarily through disinhibitory mechanisms that promote coordinated firing among local circuits. In addition to rhythm generation, they are also involved in cognitive processes such as attention, learning, and memory integration [49–51]. As such, GABAergic neurons are considered key “synchronizers” that support both oscillatory stability and information integration within the hippocampal network. ChAT⁺ neurons modulate the hippocampal network by increasing theta amplitude and suppressing SWRs. Given the critical role of SWRs in memory consolidation and replay, the “state-gating” function of ChAT⁺ neurons ensures proper rhythmic transitions during task execution and memory retrieval [48, 54, 76]. This mechanism is critically important for maintaining the temporal precision and behavioral stability required for spatial memory performance. The Glu^+^ neurons exert a relatively modest direct influence on the MS-HPC circuit. They support theta synchronization indirectly through local interactions with GABAergic neurons. Their contribution becomes more pronounced during locomotor states, where they help stabilize the rhythmic activity and facilitate the coherent hippocampal dynamics. Although these neurons are not principal oscillation generators, their modulatory role remains important for rhythm coordination during spatially relevant behaviors [55, 68].

**Fig. 1:**
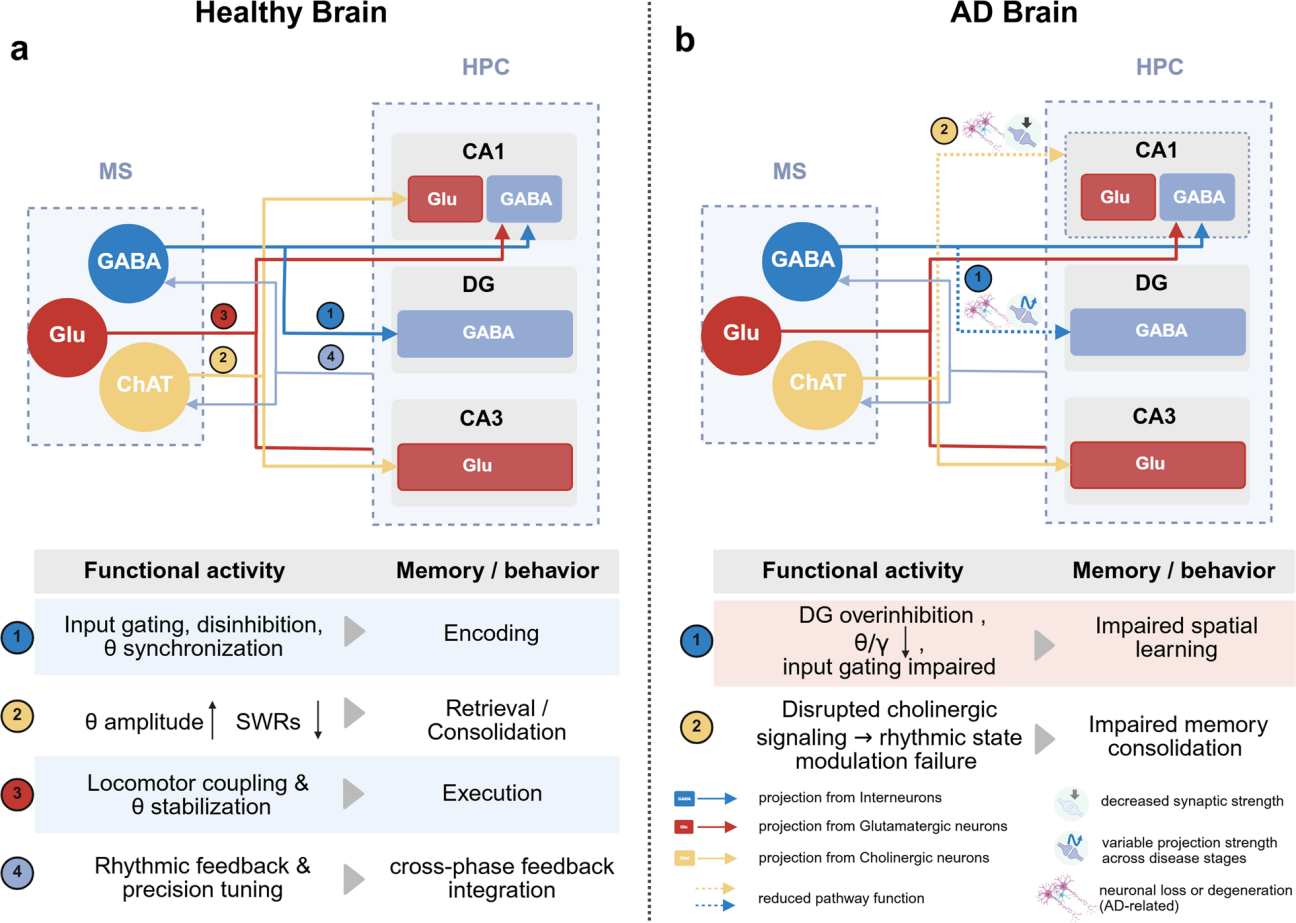
Differential organization and disruption of the medial septum-hippocampus (MS–HPC) circuitry under physiological and Alzheimer's disease (AD) conditions. **a** MS–HPC circuit in the healthy brain. The upper panel shows major projection pathways from GABAergic, cholinergic (ChAT⁺), and glutamatergic (Glu⁺) MS neurons to specific HPC subregions such as CA1, CA3, and the dentate gyrus (DG), along with partial feedback from HPC interneurons. The lower panel summarizes the functional roles of each pathway in modulating network oscillations, such as θ rhythms and sharp-wave ripples (SWRs), and in supporting different stages of spatial memory processing (encoding, retrieval, and consolidation). **b** MS–HPC circuit alterations in AD. The upper panel shows projection pathways with structural or functional impairments in AD models, such as theta rhythm desynchronization due to reduced GABAergic input to the DG and cholinergic dysfunction leading to impaired rhythmic state modulation. The lower panel outlines associated cognitive deficits, including impaired spatial learning and memory consolidation

In summary, the three major types of projection neurons in the MS play distinct but complementary roles in rhythmic regulation. The GABAergic neurons govern theta generation and synchronization, the cholinergic neurons regulate state transitions and facilitate memory retrieval processes, and the Glu^+^ neurons support oscillatory stability during movement. Through precise coordination, these neuronal populations maintain the temporal structure of hippocampal theta rhythms, ensuring efficient encoding and accurate retrieval of spatial memory (Fig. 1a). The MS–HPC pathway, anchored by GABA-mediated disinhibition and theta coordination, and modulated by cholinergic and glutamatergic inputs, serves as a fundamental rhythmic driver for spatial memory regulation.

### LS: a functional hub for spatial information integration and behavioral output

#### Anatomical organization and integration of multisource inputs

The LS is an anatomically complex structure predominantly consisting of GABAergic neurons, which account for over 90% of its neuronal population [21, 77]. These neurons widely express inhibitory markers such as PV, SST, and calretinin [77, 78]. In addition, Glu^+^ neurons are sparsely distributed and primarily localized to the ventral portion of the LS [77].

**Fig. 2:**
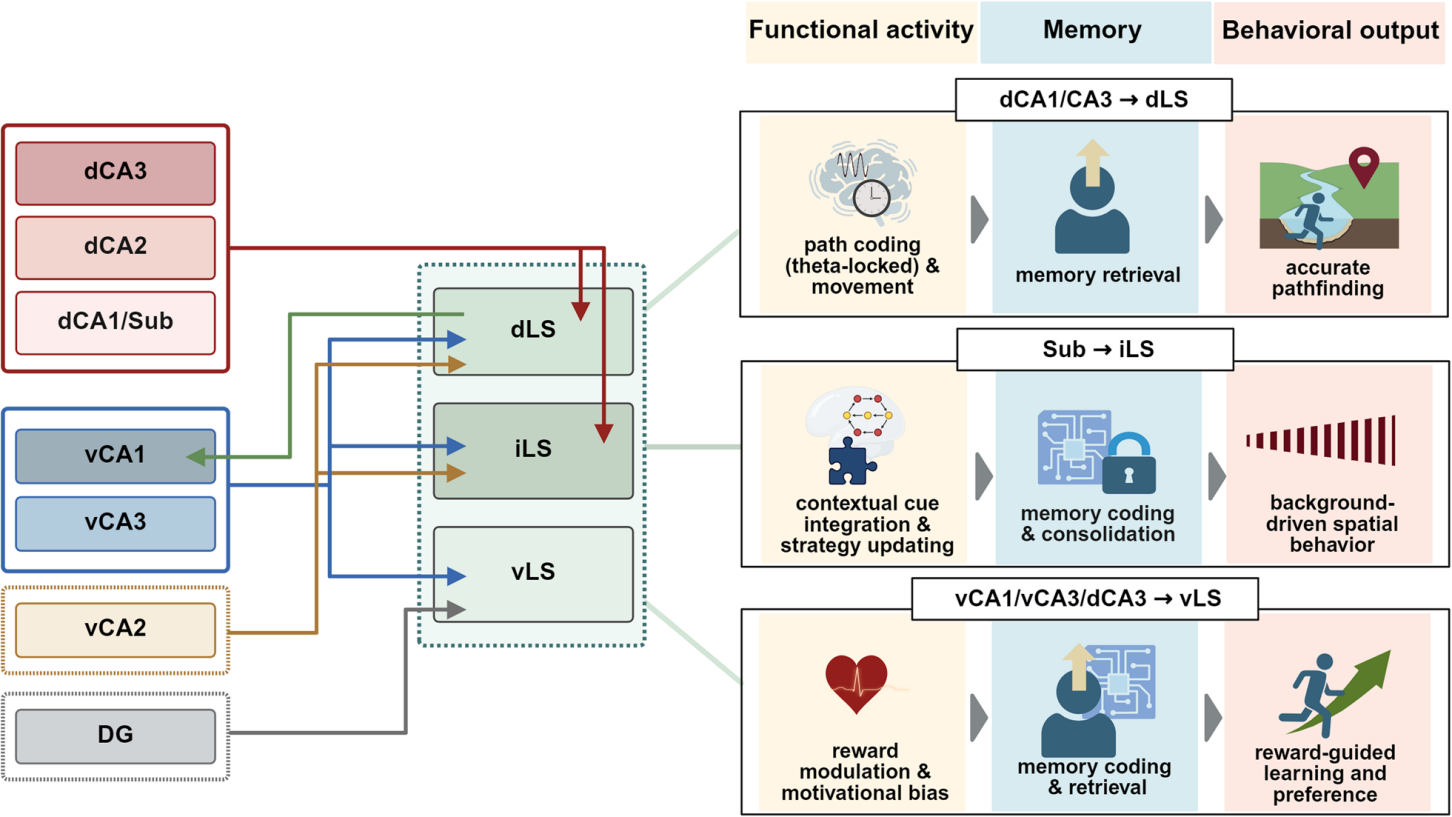
Illustration of the HPC–LS neural circuit across anatomical structures and functions. Anatomical projections from hippocampal (HPC) subregions, such as dorsal CA1 (dCA1), dorsal 441 CA3 (dCA3), ventral CA1 (vCA1), ventral CA3 (vCA3), subiculum (sub), and dentate gyrus (DG), to subregions of the lateral septum (LS), including dorsal LS (dLS), intermediate LS (iLS), and ventral LS (vLS). Functional pathways from the HPC to the LS, covering distinct types of functional activity, the associated stages of spatial memory (e.g., encoding, retrieval, consolidation), and corresponding behavioral outcomes. The diagram summarizes how multiple HPC subregions convey functionally distinct signals to LS subregions, enabling diverse aspects of spatial cognition and behavior

Situated between the MS and the lateral ventricle, the LS exhibits pronounced anatomical compartmentalization despite its predominant GABAergic composition. Along the dorsoventral axis, the LS can be subdivided into three main regions: dorsal (dLS), intermediate (iLS), and ventral (vLS). Some studies further divide these into finer subregions, including dorsomedial, dorsolateral, ventromedial, and ventrolateral areas [77, 78]. These subdivisions are based on topographically organized inputs from different hippocampal subfields and reflect a spatial correspondence along the dorsoventral axis (Fig. 2) [79, 80]. This topographic input organization not only underlies the anatomical compartmentalization of the LS, but also supports its functional diversity in spatial navigation and memory-related processes.

#### Functional specialization of LS subregions and regulation of spatial behavior

Building upon its well-defined anatomical subdivisions, the LS exhibits marked functional specialization across its subregions in the regulation of spatial behavior. Different LS subfields contribute differentially to spatial navigation, sensorimotor integration, emotional valuation, and behavioral strategy selection, reflecting a tight coupling between structure and function.

The dLS serves as a key region for spatial-motor integration. Neurons in this area are highly sensitive to locomotor variables such as speed and direction, and display predictive firing patterns, including theta phase precession, supporting path updating and temporal coordination of movement [35, 79, 81]. The vLS, through its connections with the hypothalamus and other limbic structures involved in motivational regulation, contributes to risk assessment and motivational state modulation during exploratory behavior, thereby assigning affective value to spatial decision-making [79, 82]. The caudal LS is thought to participate in context-action mapping. Its neural activity is closely associated with behavioral phase transitions and may be involved in strategy updating and behavioral sequence reorganization [78].

Behavioral studies have shown that LS lesions impair egocentric navigation based on self-motion cues, although animals may adopt alternative strategies to partially compensate for this impairment [83, 84]. Moreover, selective lesions of the dLS lead to significant impairment of mouse performance in the novel location recognition task, while the spatial recognition ability can be restored following neuronal regeneration, suggesting a critical role of the dLS in spatial memory formation [85].

Collectively, the subregions of LS cooperate to support spatial state encoding, contextual analysis, and preliminary behavioral evaluation, establishing a functional framework for regionally organized control within the spatial memory system. Whether the integration of these functional channels contributes further to goal recognition and strategy generation will be addressed in the following section.

#### Goal enhancement and strategy selection based on spatial signals

In spatial navigation behavior, the LS not only receives spatial information from the HPC but also evaluates its behavioral relevance and converts it into action-oriented strategies. Anatomically, the LS lies at the intersection of the HPC, nucleus accumbens, and hypothalamus, enabling it to integrate cognitive, contextual, and motivational signals [82, 86]. This network position supports its role in transforming spatial perception into behavioral output.

Current theories suggest that the LS plays a key role in linking spatial cues to behavioral outcomes, such as reward or punishment, acting as a critical intermediary in the perception–action pathway [87, 88]. Position-like cells in the LS, especially in its caudal region, show stable firing preferences for goal-related spatial locations. Compared to hippocampal place cells, these neurons have lower spatial resolution but stronger selectivity for goal-relevant cues, indicating a prioritization of task-relevant spatial information [89].

Many LS neurons are phase-locked to hippocampal theta rhythms, and some show enhanced firing during SWRs, suggesting involvement in memory replay or predictive simulation across regions [35, 90]. During spatial reward tasks, LS activity often precedes movement onset and is localized near reward sites, supporting its role in goal prediction and behavioral preparation [35, 82].

Together, these findings indicate that the LS not only refines hippocampal spatial representations, but also assigns value through rhythmic coordination and reward bias, converting spatial input into behaviorally relevant signals for task execution and strategy selection. This spatial-contextual-motivational integration defines the core role of LS in guiding goal-directed navigation.

### The HPC–LS circuit: contextual decoding of space for goal selection

The projection from the HPC to the LS represents a key output pathway through which spatial information is transformed into contextual reconstruction and goal-directed behavior in downstream regions. Distinct hippocampal subfields connect topographically to corresponding LS subregions: dorsal CA1/CA3 project primarily to the dorsolateral LS, conveying high-resolution path navigation signals; ventral CA regions project to the ventrolateral LS, transmitting reward- and emotion-related information; and the subiculum sends broad projections to the intermediate LS, contributing to contextual cue integration [78, 91].

Functionally, the HPC–LS pathway modulates LS neuronal bias toward target-related spatial locations, thereby enhancing the recognition of task-relevant zones and improving behavioral prediction [35]. Behavioral studies show that inhibition of this pathway disrupts reward prediction and induces disorganized navigational strategies [39, 92]. Moreover, phase-locked responses of LS neurons to hippocampal theta rhythms and SWRs events suggest a role in the stage-specific retrieval of spatial memory [88, 90, 93].

The HPC–LS pathway also participates in spatial-motivational integration. Projections from the HPC to the dLS modulate the flexibility of fear expression, and their disconnection impairs memory maintenance of food-associated target locations [93, 94]. Another study demonstrated that the CA3–dLS–hypothalamus circuit, regulated by melanin-concentrating hormone, enhances learning performance in spatial reward tasks [95].

Together, the HPC–LS pathway exerts topographically organized control over LS spatial and contextual representations, serving as a critical output mechanism for spatial memory retrieval and goal localization behavior (Fig. 2).

### Regulatory feedback from the HPC and LS to the MS

To fully understand the dynamic coordination within the MS–HPC–LS circuitry, it is essential to also consider feedback influences.

Feedback projections from the HPC to the MS suggest that hippocampal GABAergic neurons may modulate MS activity via dual mechanisms: rapid inhibition of MS GABAergic neurons and slower suppression of cholinergic neurons. And a subset of PV⁺ MS neurons both project to and receive input from the HPC, suggesting a bidirectional regulation within the septohippocampal circuit [75]. Further evidence shows that the hippocampal Glu^+^ neurons expressing dopamine D2 receptors (hD2R⁺), can project to the MS. Functional manipulation of this pathway demonstrated its involvement in regulating the accuracy of goal localization during spatial-reward association tasks, suggesting that the MS also integrates goal-directed feedback from the HPC [60].

On the efferent side, GABAergic neurons in the LS have been shown to project to the ventral CA1, supporting the anatomical basis for potential downstream modulation of hippocampal circuits [96].

In addition to hippocampal targets, trans-synaptic tracing studies have revealed that the LS neurons receiving input from hippocampal CA1 and CA3 also project to the hypothalamus and the MS, with robust synaptophysin labeling observed in the MS. These findings suggest the existence of sparse but anatomically defined LS-MS connections [33]. Beyond downstream modulation, such projections raise the possibility of a feedback loop in which LS conveys regulatory signals back to the MS. Supporting this notion, electrophysiological recordings show that the LS neurons display phase-locked firing to hippocampal theta oscillations [33], implying potential feedback influence on MS GABAergic or cholinergic neurons involved in rhythm generation or behavioral state control [39].

Although the precise mechanisms of this feedback remain to be fully elucidated, its potential role as a modulatory loop within the MS–HPC–LS circuitry suggests that it may contribute to rhythmic coordination and the dynamic updating of spatial memory states.

Beyond the established role in spatial cognition, the MS–HPC–LS circuitry also serves as a highly coordinated neural network involved in various physiological processes. Studies have shown that different subcircuits, particularly the interactions between MS and HPC and between HPC and LS, contribute to the regulation of hippocampal theta oscillations and maintenance of the wake-sleep cycle (primarily via the MS-HPC pathway), as well as the integration of emotional states and modulation of stress responses (primarily via the HPC-LS pathway). Specifically, the MS modulates hippocampal theta rhythms through rhythmic neuronal activity, contributing to synchronized oscillations during wakefulness and REM sleep [97, 98]; the LS serves as a hub for emotional regulation, modulating anxiety-related behaviors and social interactions [79]; and the HPC, in addition to its role in spatial information processing, is also involved in the integration of cognitive states and emotional responses [99]. In the following, we will focus on the role of this circuitry in spatial memory impairment and potential therapeutic strategies in AD.

## Dysfunction of the MS–HPC–LS circuitry in early AD

**Fig. 3:**
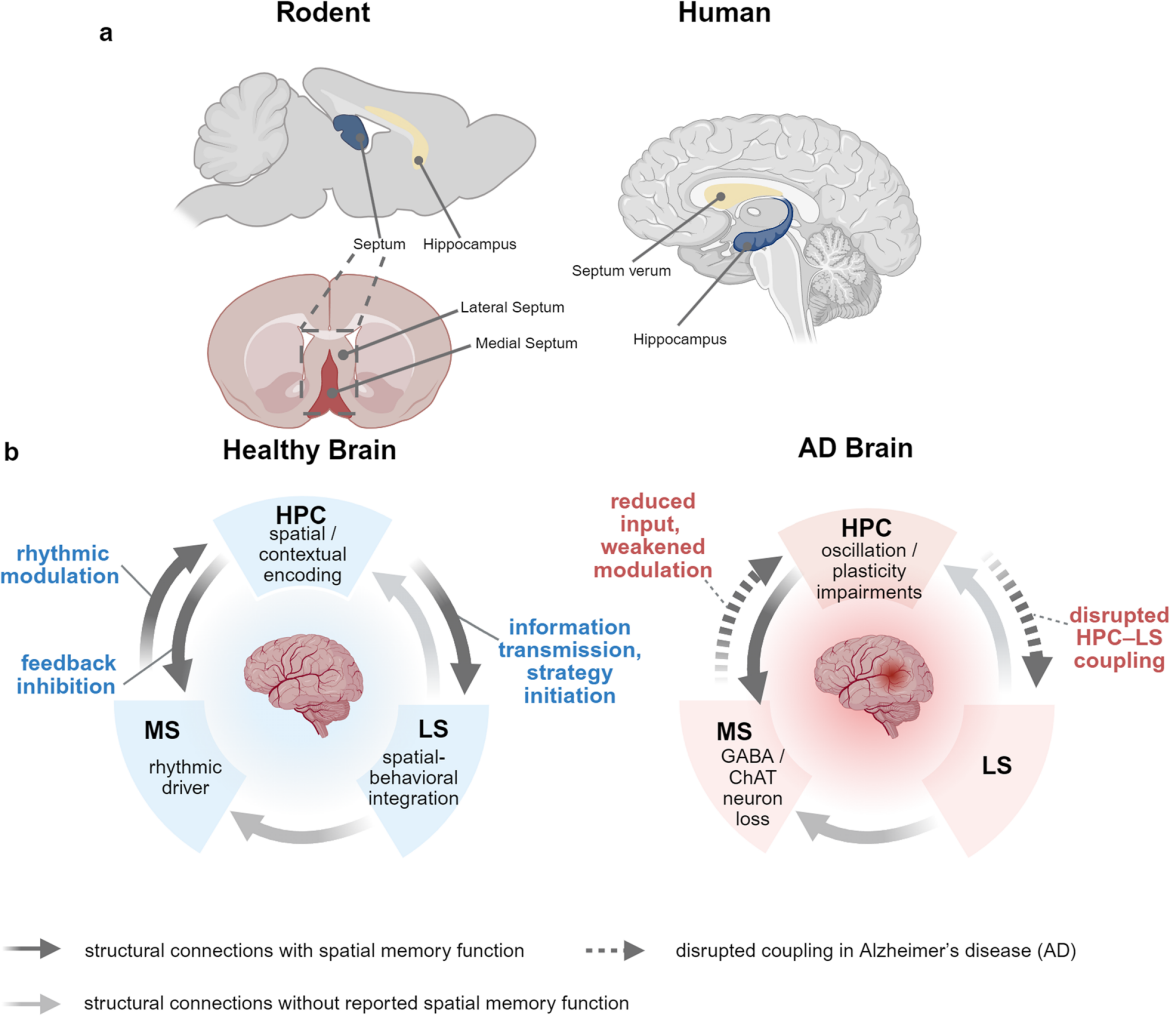
Cross-species anatomy and AD-related functional breakdown of the MS–HPC–LS circuitry. **a** Schematic representation of the medial septum (MS), hippocampus (HPC), and lateral septum (LS) in the rodent and human brains. In rodents, the MS and LS are anatomically distinguishable, located along the midline and lateral wall of the septal area, respectively. In humans, the homologous region, referred to as the septum verum, is situated anterior to the HPC and is thought to include medial and lateral divisions, although these subdivisions are not clearly delineated anatomically in the human brain and thus not explicitly labeled in this illustration [40]. **b** Functional and structural differences in the MS–HPC–LS circuitry in healthy versus AD conditions. In healthy brains, the MS, HPC, and LS are interconnected via directional anatomical projections, forming a functional circuit that supports spatial memory processing. In AD, both the structural integrity and inter-regional communication of this circuitry are compromised, contributing to spatial disorientation and cognitive decline

Early AD is associated with reduced structural connectivity in the default-mode network [100]. Recent studies have highlighted the importance of the entire Papetz circuit in memory and spatial navigation deficits. This network is essential for both episodic memory formation and allocentric navigation, and exhibits progressive dysfunction during initial AD pathogenesis [101]. Impairments in the hippocampal GABAergic neural network are significantly correlated with spatial memory deficits in early-stage AD. Dysfunction within this system results in hippocampal hyperactivity, exacerbating the memory and cognitive decline in AD patients [102]. Additionally, the entorhinal cortex and its connections to the DG and CA3 regions of the HPC are particularly vulnerable in AD, with their structural and functional changes potentially predicting disease progression [103]. These findings emphasize the importance of the HPC-related circuit in AD pathophysiology and symptomatology. Under pathological conditions, disruptions of the circuit connecting the MS, HPC, and LS could result in a decline in spatial memory function. Exploring the organization and activity of this circuit during the onset of early AD may help identify initial pathological alterations associated with the disease (Fig. 3).

### Aβ/tau-driven progressive disintegration of the MS–HPC circuit connectivity

During the early pathological stage in the 3 × Tg-AD mouse model, GABAergic projections from the MS to the DG are significantly reduced [59]. Degradation of GABAergic synapses in AD is associated with a loss of PV^+^ neurons, and these changes are particularly pronounced in the MS–HPC circuit [104]. Aβ and tau pathology may lead to damage to the MS–HPC GABAergic neurons. The hAPP_SwInd_ mouse model (which shows considerable Aβ accumulation) exhibits early-onset decrease of strength of GABAergic MS–HPC projections, which is not caused by neuronal loss, but by reductions in the number and the complexity of axon terminals. Phosphorylated tau may lead to further loss of inputs [105]. These findings shed light on the dynamics of GABAergic neurons during AD and its potential compensatory mechanisms. Furthermore, in 4.5-month-old 5 × FAD mice, the SHS (MS–HPC–MS) circuit begins to deteriorate before the onset of cognitive impairment, and this degenerative process is accelerated in 14-month-old 5 × FAD mice [106]. Monosynaptic rabies tracking revealed significantly decreased input connections from the MS-DBB to excitatory CA1 neurons in APP-knockin mice compared to WT mice [107]. These findings provide topological evidence for the degradation of interconnections between the MS and HPC.

An increasing number of anatomical and functional studies have demonstrated that cholinergic neuronal loss in the MS occurs in both healthy aging and AD brains [108], and that cholinergic innervation in the HPC is severely affected [109–111]. It is notable that these synaptic injuries begin to occur before Aβ aggregation and accelerate neural circuit dysfunction, ultimately leading to cognitive decline [112, 113]. Furthermore, it has been suggested that extensive impairment of synaptic function is at the root of neural circuit imbalance [114].

### Disrupted MS–HPC circuit dynamics underlie the spatial memory deficits in AD

Clinical investigations have consistently demonstrated prominent spatial learning impairments in patients with AD [115]. Animal studies have provided insights into the neural mechanisms of this deficit, highlighting the MS as a critical upstream node in early hippocampal dysfunction [107]. In multiple transgenic mouse models, including APP/PS1, 3 × FAD, and J20, early disturbances in the MS–HPC circuit have been observed, characterized by diminished GABAergic tone and reduced hippocampal theta and gamma oscillatory power [116–118]. These findings suggest that disruption of the MS–HPC circuit may underlie the spatial cognitive deficits in early-stage AD. To test this hypothesis, acute AD mouse models were established by intraseptal injection of Aβ1-40, which induced marked suppression of hippocampal theta rhythms and disruption of cholinergic and Glu^+^ activity governed by GABAergic neurons. In vivo local field potential (LFP) recordings, together with behavioral tests such as the Morris water maze and Y-maze, confirmed spatial memory impairments [119]. Moreover, in J20 mice with Aβ deposition, 40 Hz optogenetic stimulation of the MS PV^+^ neurons restored hippocampal slow gamma oscillation amplitude and phase-amplitude coupling, and rescued spatial memory performance [21]. These findings support the view that the MS–HPC circuit dysfunction is an early and modifiable pathological feature of AD.

In addition to amyloid-driven toxicity, tau pathology has been implicated in the MS–HPC circuit instability. In mice overexpressing human full-length tau (hTau) and other tauopathy mouse models, tau deposition in the MS disrupts the theta synchrony between MS and hippocampal CA1 neurons, thereby impairing memory consolidation processes [120]. Chemogenetic activation of cholinergic projections from the MS and ventral diagonal band to the HPC ameliorated spatial memory deficits in APP/PS1 mice [121], suggesting a potential therapeutic avenue targeting septohippocampal cholinergic integrity.

Beyond early MS–HPC disruptions, GABAergic overactivation within the DG has also been implicated in AD-related memory decline. In late-stage mouse models, sustained hyperactivity of DG interneurons suppresses the granule cell excitability. Although MS-derived GABAergic projections to DG increase in an apparent compensatory manner, this fails to restore the local excitation–inhibition balance, ultimately leading to reduced granule cell activity and worsening of spatial memory performance [59]. This progressive circuit-level dysfunction and its impact on spatial memory are illustrated in Fig. 1b.

### HPC–LS functional decoupling precedes cognitive deficits in early AD

Functional decoupling between the HPC and the LS appears to be an early pathological feature of AD. A study in J20-AD transgenic mice showed that the functional connectivity between hippocampal CA1 and the LS is already disrupted before the onset of measurable cognitive deficits. By simultaneously recording the single-neuron activity of the LS and the LFP activity from the hippocampal CA1 region, researchers found reduced LS-CA1 coupling, particularly within the theta frequency range, indicating an early disruption in rhythmic coordination between the HPC and the LS. This finding suggests that the AD-related circuit dysfunctions may begin long before measurable behavioral impairments, and the aberrant functional connectivity between the HPC and the LS may contribute to the cognitive deficits observed in AD [25].

## Synaptic alterations induce the MS–HPC–LS circuitry impairment in AD

The MS–HPC–LS neural network plays a role in spatial memory and impairment of this neural network causes spatial memory deficits in AD. However, the mechanisms underlying the impairment of this neural network remain unclear. Impairment of the MS–HPC circuit in AD manifests as reduced projection density, synaptic loss, further neuronal death and loss due to synaptic damage, and a significant reduction in the HPC-LS neural network activity, all of which originate at the synaptic transmission level. In addition, synaptic loss and synaptic injury are also considered to be the best correlated factors for cognitive deficits in AD patients. In the following, we will discuss the mechanism of MS–HPC–LS neural network damage from the perspective of synaptic transmission.

### Synaptic changes and loss in AD

Synapses are a fine structure through which neurons are connected to form a dense neural network and transmit electrical and chemical signals. Synaptic plasticity involves the alterations of the strength of connections between neurons, including functional changes (e.g., neurotransmitter levels, excitability, electrical activity, or postsynaptic receptors) and structural changes (e.g., growth in dendritic spines, branches, axon terminals, or synapses) [122]. Physiologically, synaptic plasticity mainly refers to changes in the synaptic transmission efficiency. Presynaptic plasticity involves modifications in the neurotransmitter release probability, while postsynaptic plasticity entails alterations in receptor density or subtype composition on the postsynaptic membranes [123]. Synaptic plasticity is the core of neural network change. Although the precise mechanisms behind synaptic loss and dysfunction in various diseases remain poorly understood, evidence suggests that a decrease in synaptic activity and density is one of the earliest events in many CNS disorders [124]. During the progression of AD, the anatomical structure of the MS–HPC–LS circuitry is significantly altered and leads to varying degrees of spatial memory deficits, affecting different stages of spatial memory. Therefore, understanding the mechanisms underlying the synaptic deficits can help restore spatial memory deficits caused by impairment of the MS–HPC–LS circuitry.

### Aβ and tau oligomers drive synaptic vulnerability in AD

Aβ and tau are the main pathogenic factors of AD, which disrupt synaptic plasticity and mediate synaptic toxicity through different mechanisms. Soluble oligomers of Aβ and tau can spread in different areas of the brain, directly leading to synaptic dysfunction and loss. The presence of Aβ deposition or tau protein abnormalities in HPC, MS, and LS regions at specific months of age in different AD mouse models [125–128] highlights the potential vulnerability of this circuit in early stages of AD. Deletion of tau protein leads to severe defects in LTP but not long-term depression (LTD) in the CA1 region of the HPC. This may result from disrupted synaptic targeting of Fyn kinase, which weakens the NMDAR–PSD95 signaling required for LTP induction and the AMPAR trafficking underlying LTP expression, whereas LTD relies less on these mechanisms and is therefore relatively preserved [129, 130]. Reducing the endogenous tau levels prevents synaptic dysfunction, mediated by changes in postsynaptic molecules, in an AD mouse model [131]. This suggests that the role of tau in synaptic function may differ between physiological and pathological conditions. In AD, Aβ and tau protein oligomers accumulate extracellularly and in synaptic cytoplasm, respectively, in regions with massive synaptic loss. Notably, their clearance has been shown to restore synaptic function. In addition, synaptic deficits are a characteristic of tauopathies [132].

In recent years, considerable progress has been made in elucidating how Aβ and tau oligomers contribute to synaptic dysfunction in AD, particularly during early stages when synaptic plasticity and neural circuits begin to exhibit vulnerability. Zhou et al. integrated spatial transcriptomic and single-nucleus RNA sequencing data from both 5 × FAD transgenic mice and human AD brain tissues, revealing that the inhibitory GABAergic neurons in cortical regions display marked transcriptional dysregulation even before widespread Aβ plaque deposition, suggesting that the circuit-level impairments may precede classical pathological hallmarks [133]. In vitro, tau protein undergoes liquid–liquid phase separation (LLPS) and abnormally accumulates within reconstituted postsynaptic density-like condensates, disrupting glutamate receptor anchoring and synaptic signaling, which leads to synaptic destabilization and dysfunction. In vivo studies further provide indirect evidence supporting LLPS-associated tau toxicity at the synaptic level [134, 135]. Additionally, two-photon in vivo imaging in APP23 × PS45 transgenic mice showed that early clearance of soluble Aβ monomers reverses neuronal hyperexcitability and improves synaptic function. This suggests that the Aβ-induced synaptic impairment may be partially reversible and highlights a potential therapeutic window for early intervention at the circuit level [136]. Collectively, these findings expand our understanding of Aβ- and tau-related toxicity and emphasize the central role of synaptic dysfunction in disrupting the MS–HPC–LS circuitry. Importantly, such damage is not solely driven by Aβ and tau aggregation but also involves an imbalance in glutamatergic and GABAergic signaling, along with non-cell-autonomous processes such as neuroinflammation and oxidative stress [137]. These findings highlight the dynamic and multifactorial nature of Aβ- and tau-induced synaptic injury, and underscore their critical role in initiating synaptic destabilization within AD-vulnerable circuits such as the MS–HPC–LS pathway. In the following sections, we will discuss downstream mechanisms, including changes of receptor signaling and non-cell-autonomous contributors.

### Glutamate receptors as a mechanism by which Aβ and tau cause synaptic loss

**Fig. 4:**
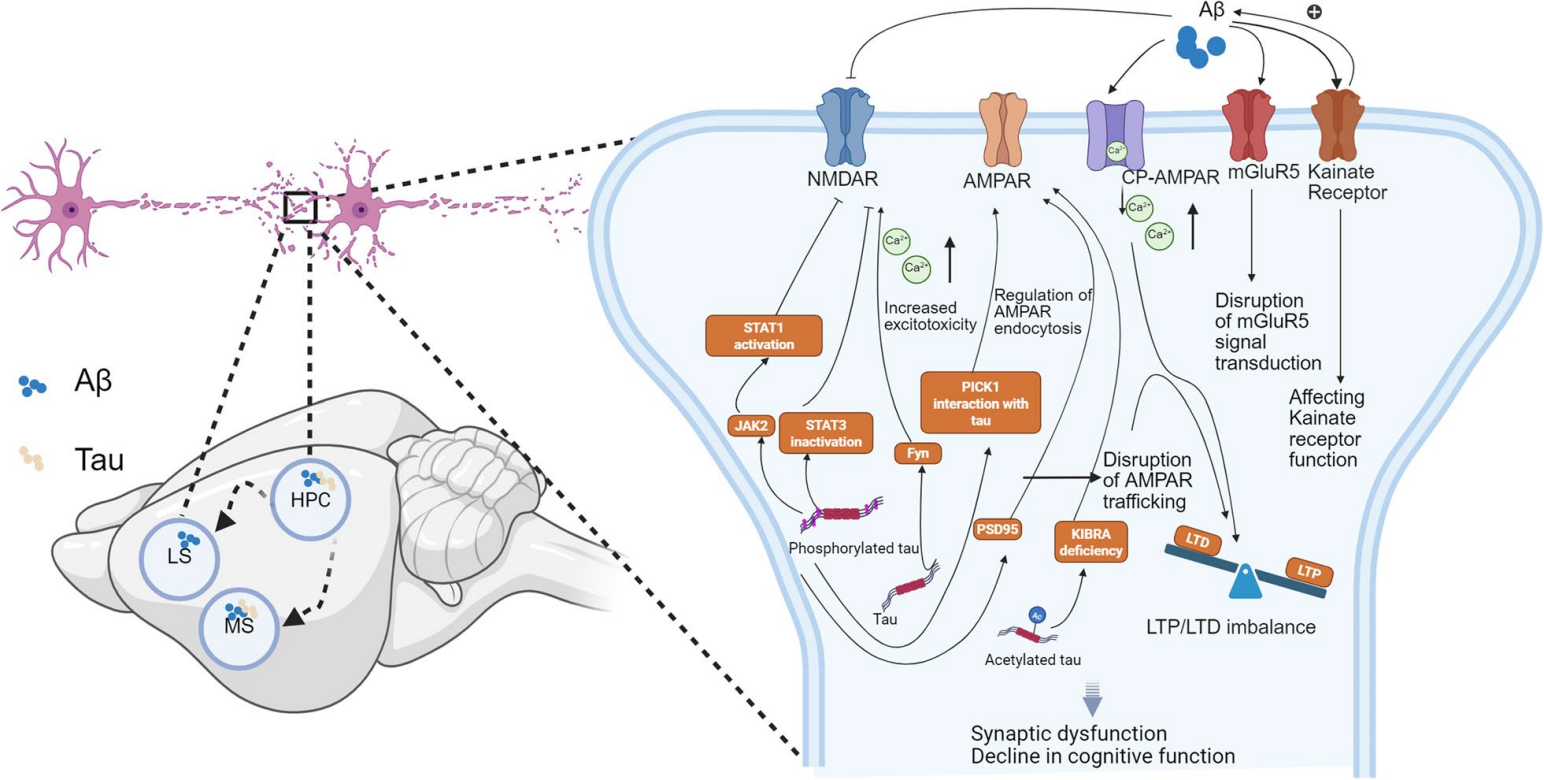
Potential mechanism underlying the impairment of the MS–HPC–LS neural circuitry from the perspective of synaptic dysfunction caused by Aβ or tau. Left, neurodegenerative changes are detected in all three brain regions of the circuit at different stages of AD progression. Aβ first appears in the hippocampus (HPC) of 2-month-old 5 × FAD mice [125], while both the medial septum (MS) and the lateral septum (LS) show intracellular Aβ plaque accumulation by 4.5 months in the same model [106]. Tau pathology is observed in the HPC of 2.5-month-old rTg4510 mice (tau P301L) [127], whereas in Tg601 mice expressing wild-type human tau, tau accumulation in the MS is evident at ~ 6 to 8 months of age (128). Dashed arrows represent possible Aβ and tau propagation pathways. The negative effects of Aβ and tau on synaptic damage may be mainly due to their toxic effects on glutamate synaptic receptors. Right, synaptic-level mechanisms by which Aβ and tau exert toxicity on various glutamate receptor subtypes, including NMDA receptor (NMDAR), AMPA receptor (AMPAR), CP-AMPAR, metabotropic glutamate receptor 5 (mGluR5), and kainate receptor. Aβ promotes excitotoxicity and interferes with AMPAR/NMDAR trafficking, while tau, through phosphorylation and acetylation, modulates downstream effectors such as Fyn, postsynaptic density protein 95 (PSD95), protein interacting with C-kinase 1 (PICK1), and kidney and brain expressed protein (KIBRA). These disruptions lead to AMPAR endocytosis, impairment of mGluR5 signaling, and an imbalance between long-term potentiation (LTP) and long-term depression (LTD), collectively resulting in synaptic dysfunction and cognitive decline

The MS, HPC, and LS regions all express glutamate receptors. As one of the main input areas to the HPC, the MS regulates neural activity in the HPC by releasing glutamate [138]. Dysregulation of hippocampal glutamatergic signals is closely related to the impairment of memory and learning functions [139]. As the main downstream nucleus of the HPC, the LS receives extensive glutamatergic neuronal input from the HPC. The main glutamate receptors involved in this neural circuit are NMDA receptors (NMDARs), metabotropic glutamate receptors (mGluRs), AMPA receptors (AMPARs), and kainate receptors (KARs), which are essential for maintaining normal cognitive function [140–142]. In the context of AD, glutamate receptors in the MS–HPC–LS neural circuit may be a key point for therapeutic intervention. The accumulation and spread of Aβ and tau proteins may disrupt synaptic plasticity by affecting the function of these receptors, thereby affecting cognitive function (Fig. 4).

#### Mechanisms of synaptic dysfunction caused by Aβ

Aβ oligomers cause rapid internalization of synaptic NMDARs, leading to dendritic spine loss and impaired LTP [143]. They also alter the conformation of the C-terminal domain of NMDAR, weakening its interaction with protein phosphatase 1, a process that can be rescued by overexpression of postsynaptic density protein 95 (PSD-95) [144]. Further, Aβ enhances clustering of the NR2B subunit via integrin β1 and classical PKC signaling, increasing Ca^2+^ influx and destabilizing the NMDAR-mediated synaptic activity [145]. Importantly, Aβ also activates extrasynaptic NMDARs, which suppress CREB signaling and impair the transcriptional pathways essential for memory consolidation [146]. These effects render NMDARs a central node in Aβ-induced excitotoxicity.

Aβ also disrupts AMPAR trafficking, particularly through upregulation of calcium-permeable AMPARs (CP-AMPARs) that lack the GluA2 subunit [147–149]. This alteration increases Ca^2+^ permeability and disrupts homeostatic synaptic plasticity. Recent findings suggest that this process involves breakdown of the diffusion-trap mechanism that normally stabilizes AMPAR localization, promoting aberrant CP-AMPAR anchoring and receptor overactivation [149]. GluA3-containing AMPARs are specifically required for Aβ-induced toxicity, as genetic deletion of GluA3 protects against synaptic and cognitive deficits [150]. In vivo imaging further confirms that the CP-AMPAR insertion promotes dendritic spine shrinkage and synaptic pruning, and impairs excitatory transmission [151]. These findings highlight the contributions of AMPAR subtype imbalance and trafficking defects to AD-related synaptic degeneration.

mGluR5 is also emerging as a critical mediator of Aβ toxicity. Aβ oligomers interact with the mGluR5-PrPC (cellular prion protein) complexes, disrupting downstream signaling required for synaptic plasticity [152]. Notably, a recent positron emission tomography (PET) imaging study in human subjects revealed that the mGluR5 level is positively correlated with synaptic density and hippocampal volume and negatively correlated with Aβ burden [153]. This suggests that mGluR5 may serve not only as a functional mediator of synaptic impairment but also a potential early biomarker of circuit vulnerability in AD.

KARs may also be affected in AD, although their roles are less defined. KAR dysfunction in AD is supported by postmortem findings showing reduced GluK1-3 subunit immunoreactivity in hippocampal and parietotemporal regions. Recent evidence shows a link between GluK1-dependent impairment of inhibitory circuit development and altered network synchronization [154, 155]. Modulation of KAR activity has been proposed as a potential therapeutic strategy to restore synaptic and network balance in AD models [156].

#### Mechanisms of synaptic dysfunction caused by tau

Tau interferes with the transport and synaptic anchoring of glutamate receptors, including both AMPARs and NMDARs, thereby disrupting synaptic function [157]. Recent studies have demonstrated that hTau impairs synaptic plasticity by downregulating NMDAR expression through the JAK2 (Janus kinase 2)/signal transducer and activator of transcription 1 (STAT1) pathway. Conditional knockout of STAT1 using AAV-Cre or expression of dominant-negative Y701F-STAT1 rescues the hTau-induced suppression of NMDAR expression and improves synaptic function and memory in mice [158]. Similarly, overexpression of human P301L mutant tau inactivates STAT3, suppressing NMDAR expression. Restoring STAT3 function alleviates the tau-induced synaptic and cognitive deficits by increasing NMDAR expression, underscoring a transcriptional control mechanism in tau pathology [159]. In parallel, tau modulates NMDAR excitotoxicity by promoting mislocalization of Fyn kinase to the postsynaptic membrane, which enhances NR2B-associated signaling and Ca^2+^ overload [130, 131].

Tau also disrupts AMPAR function through multiple mechanisms. At the postsynaptic terminal, tau facilitates the interaction between GluA2 and PICK1 (protein interacting with C-kinase 1), promoting AMPAR endocytosis and contributing to hippocampal LTD [160, 161]. Aberrant tau phosphorylation reduces the transport of GluA1 and GluA2/3 to the PSD-95 scaffold, while acetylated tau impairs the KIBRA (kidney and brain expressed protein)-mediated actin polymerization, destabilizing AMPAR anchoring [162, 163]. These impairments compromise synaptic plasticity and memory. Moreover, tau has multivalent interactions with PSD-95, constraining postsynaptic density dynamics and potentially enhancing NMDAR hyperactivity [134]. Together, these findings highlight tau as a multifaceted disruptor of glutamatergic synapse integrity through both transcriptional and structural pathways.

### Changes of synaptic transmission of GABAergic neurons in the MS–HPC–LS circuitry

In AD, dysfunction of GABAergic neurons is a central factor in the impairment of the MS–HPC–LS circuitry. Emerging evidence suggests that the GABAergic deficits may arise during early AD stages and could be linked to complement-mediated synaptic loss [164]. Accumulation of Aβ and tau disrupts GABAergic function, leading to an excitation/inhibition imbalance that subsequently impairs spatial memory and learning [165]. The balance between GABAergic and glutamatergic systems is fundamental for neuronal circuit function. In AD, decreased GABA levels lead to failure in counteracting glutamatergic excitation, thereby exacerbating excitotoxicity [166]. Recent studies increasingly highlight that GABAergic dysfunction plays a central role in early AD-related circuit impairment, particularly in the MS–HPC–LS network.

#### Effects of Aβ deposition on GABAergic transmission

Aβ accumulation disrupts inhibitory neurotransmission through multiple mechanisms, including indirect interference with GABA_A_ receptor expression and localization. Studies have reported downregulation of GABA_A_ receptor subunits and altered postsynaptic distribution in AD brains, possibly linked to Aβ-induced cellular stress, inflammatory responses or impaired receptor trafficking [167, 168]. These changes weaken the inhibitory synaptic transmission, leading to uncontrolled excitatory input, resulting in local network hyperactivity, excitotoxicity and neuronal damage [169]. The MS–HPC–LS circuitry, which relies on rhythmic inhibitory input, undergoes progressive destabilization that disrupts both the encoding and consolidation of spatial memory [165].

#### Tau pathology and dynamic changes of GABA receptors

Abnormal hyperphosphorylation of tau and neurofibrillary tangle formation are key pathological features of AD and have significant toxic effects on GABAergic neurons. Tau pathology causes a loss of GABAergic interneurons, contributing to impairments of synaptic plasticity and memory in AD models [170]. Abnormal tau accumulation disrupts the microtubule structure and cytoskeletal stability, interfering with axonal transport and synaptic localization of GABA_A_ receptors [171]. Tau may also directly interact with GABA_A_ receptors, altering their conformation and function, further exacerbating synaptic dysfunction [172]. These mechanisms collectively diminish the inhibitory control within the MS–HPC–LS network, compromising neuronal rhythmicity and information transmission, ultimately impairing spatial navigation and memory formation.

#### Alterations in GABA metabolism and transport

In addition to receptor-level changes, disturbances in GABA metabolism and reuptake contribute to AD pathology. GAD, the enzyme responsible for GABA synthesis, is consistently downregulated in AD patient and animal model brains, reducing GABA availability and the inhibitory signaling [173, 174]. Concurrently, expression of GABA transporter-1 is also reduced, slowing GABA clearance from the synaptic cleft [175]. Although extracellular GABA levels may paradoxically rise, such nonspecific extrasynaptic diffusion disrupts normal information coding and exacerbates the excitation/inhibition imbalance, further impairing cognitive function [168].

### Other contributors to MS–HPC–LS circuitry dysfunction: neuroinflammation and oxidative stress

Beyond Aβ and tau pathologies, neuroinflammation and oxidative stress may also contribute to early dysfunction of the MS–HPC–LS circuitry in AD. Numerous studies have focused on the HPC and MS within this pathway, showing that these pathological processes can disrupt the local synaptic architecture and network coordination, ultimately impairing the cognitive function of the entire circuitry.

Microglia, the resident immune cells of the CNS, are highly responsive to Aβ plaques and hyperphosphorylated tau aggregates. Upon activation, they release a range of pro-inflammatory cytokines (such as IL-1β, TNF-α, IL-6), reactive oxygen species (ROS) and nitric oxide, leading to chronic neuroinflammation, synaptic loss and neuronal dysfunction within the HPC and MS [176–178]. Single-cell transcriptomic studies have further identified disease-associated microglia (DAM) subpopulations that accumulate in the HPC of AD mouse models and human AD brains, promoting aberrant synaptic pruning and structural remodeling of local neural circuits [179, 180]. Although microglia are considered the main mediators of neuroinflammation in AD, astrocytes and vascular dysfunction may also contribute to neuroinflammation.

Mitochondrial dysfunction represents a major source of oxidative stress in AD, particularly within hippocampal neurons [181, 182]. Aβ and tau impair mitochondrial dynamics, bioenergetics and calcium homeostasis, resulting in excessive ROS production and subsequent oxidative damage to lipids, proteins and nucleic acids. This cascade ultimately compromises synaptic integrity and neuronal survival [183]. A recent study further demonstrated that enhancing the NAD⁺ level via administration of nicotinamide mononucleotide can effectively restore mitochondrial function, reduce oxidative stress and mitigate synaptic and neuronal loss in the HPC of AD models [184], suggesting the therapeutic potential of targeting mitochondrial metabolism. Given that mitochondrial dysfunction and oxidative stress have been confirmed in multiple brain regions [185], these pathological mechanisms may also disrupt the functional integrity of the MS–HPC–LS neural circuitry.

Furthermore, there is a bidirectional amplification loop between inflammation and oxidative stress: ROS can further promote microglial activation, while sustained inflammation exacerbates mitochondrial damage. This vicious cycle is proposed to be a key mechanism underlying the progressive disconnection and dysfunction of the MS–HPC–LS circuitry observed in animal models and early-stage AD patients [186].

Neuroinflammation and oxidative stress ultimately disrupt the structural and functional integrity of the MS–HPC–LS circuitry, impairing the neural basis of spatial memory [187]. Animal studies suggest that the microglia-mediated inflammation and the ROS-induced mitochondrial damage suppress synaptic plasticity and neurogenesis in the hippocampal DG and CA1 regions, thereby compromising spatial information encoding. Additionally, inflammation and oxidative stress may impair the pacemaker function of the medial septal neurons, reduce theta rhythm generation, and weaken the MS–HPC synchrony that plays a pivotal role in spatial navigation and memory consolidation [188, 189]. Thus, the synergistic effects of inflammation and oxidative stress are considered critical contributors to the onset and progression of spatial memory deficits in AD.

In summary, synaptic dysfunction and degeneration emerge as the primary pathological process underlying the MS–HPC–LS circuitry impairment and spatial memory loss in AD. A combination of Aβ/tau aggregation, neuroinflammatory activation and oxidative stress contribute to the synaptic dysfunction and degeneration.

## Clinical translation of MS–HPC–LS circuitry modulation

Although current research on the MS–HPC–LS circuitry has primarily relied on animal models, recent clinical and neuroimaging studies have begun to reveal the potential importance of key nodes within this circuitry for spatial navigation and memory function in humans. PET studies have shown decreased cholinergic activity in the MS of early-stage AD patients, and this reduction predicts the cortical spread of AD pathology [190], suggesting that MS dysfunction may be closely related to early cognitive deficits. While direct structural and functional imaging evidence for the MS/LS–HPC connectivity in humans remains limited, extensive animal studies have clearly demonstrated strong anatomical connections between these regions and their key regulatory roles in spatial learning and memory [39, 59]. In addition, human neuroimaging studies have revealed reduced functional and structural connectivity between the HPC and medial temporal cortex regions (e.g., parahippocampal gyrus) in early AD, indirectly supporting the concept of spatial and memory network dysfunction centered around the HPC [191].

### Neuromodulation of the MS–HPC–LS circuitry

While no existing therapies can specifically target the MS–HPC–LS circuit, current treatments such as deep brain stimulation (DBS), transcranial magnetic stimulation (TMS), and other neuromodulation techniques have demonstrated potential in the treatment of neurodegenerative diseases and cognitive impairments. These approaches may indirectly influence functional recovery of the MS–HPC–LS circuitry. For example, DBS has been employed to improve cognitive function in AD patients. Studies suggest that DBS modulates electrical activity in specific brain regions, potentially altering the dynamics of neural circuits [192]. DBS in the MS restores spatial memory after cholinergic lesions through increased cholinergic activity and neurogenesis in the HPC [193]. While these studies were not optimized for the MS–HPC–LS circuitry specifically, they provide a critical theoretical foundation for circuit-based repair strategies. Additionally, TMS, as a non-invasive neuromodulation technique, has been used to regulate subcortical neural activity, improving cognitive function and memory [194]. Repetitive TMS has shown potential in ameliorating episodic memory deficits in amnestic mild cognitive impairment by modulating hippocampal subregions and their functional connectivity [195]. Further research on how these methods stimulate or modulate specific neural circuits may provide new insights into the early treatment of AD. Transcranial direct current stimulation (tDCS) modulates the excitability of cortical neurons by applying low-intensity direct current. Given that the HPC is one of the most affected regions in AD, recent studies have explored targeted neuromodulation of hippocampal circuits. tDCS has been shown to improve cognitive function, particularly by enhancing hippocampal plasticity and neural connectivity, thereby benefiting spatial memory [196]. However, the clinical application of tDCS faces several limitations, including low stimulation depth, high inter-individual variability in treatment response, and a lack of consensus on optimal stimulation parameters [197]. Other neuromodulation techniques, such as DBS and TMS, may offer alternative approaches. These methods have the potential to regulate the MS–HPC–LS circuitry, which is closely linked to spatial memory and cognitive function. By modulating hippocampal activity, DBS or TMS might indirectly enhance the connectivity between the MS and LS, thereby promoting the functional recovery of this critical neural circuit. Although these treatment methods have shown some efficacy in clinical applications, more studies are needed to reveal how they facilitate cognitive recovery through the MS–HPC–LS circuitry. Future research could focus on the development of circuit-specific neuromodulation techniques and precise regulation of the function of the MS–HPC–LS circuitry.

### Clinical challenges and emerging improvements

Despite the potential of DBS and TMS in modulating neural circuit dysfunction in AD, significant clinical challenges remain. Current DBS studies primarily target the fornix or nucleus basalis of Meynert [198, 199], with limited exploration of the MS and LS. The deep location and the high anatomical variability of the LS increase the risks of targeting errors and suboptimal outcomes [200]. Similarly, TMS is limited by poor penetration depth, restricting its ability to modulate deep circuits like the MS–HPC–LS curcuit [201, 202].

Translation of findings from animal models to human patients is facing challenges. While stimulation of the MS–HPC circuit in rodents can restore theta rhythms and cognition [203], there may be compromised responsiveness in AD patients due to their cortical atrophy and network remodeling. Anatomical and electrophysiological variabilities among individuals also limit the reproducibility and generalizability of current stimulation strategies [204, 205].

Another challenge is the lack of dynamic closed-loop modulation. The MS–HPC–LS network naturally operates through phase-locked interactions during hippocampal theta oscillations. Conventional DBS and TMS provide continuous open-loop stimulation, which may desynchronize network activity and reduce the efficacy [206, 207].

Safety and standardization remain additional concerns. DBS carries risks of hemorrhage and infection, particularly in elderly AD patients [208]. TMS has a better safety profile but lacks standardized stimulation protocols and patient selection guidelines, limiting clinical adoption and reproducibility [209].

Emerging technologies offer new directions. Closed-loop DBS, which adjusts stimulation in real time based on neural activity, has been clinically implemented in Parkinson’s disease and remains under preclinical exploration in AD models [210, 211]. However, real-time biomarkers for the MS–HPC–LS circuit feedback remain undeveloped, keeping closed-loop systems largely experimental [212]. Navigated TMS (nTMS) improves spatial precision but still lacks direct control of deep circuit activity [213]. Recent studies have also highlighted that closed-loop DBS and nTMS face significant limitations in AD, particularly in identifying real-time biomarkers and achieving effective modulation of deep brain circuits [214]. Future research should focus on developing biomarkers specific to the MS–HPC–LS circuit and enhancing the capability of nTMS to influence subcortical regions, which may ultimately advance the clinical translation of neuromodulation strategies in AD.

In summary, neuromodulation holds promise for circuit-level intervention in AD, yet substantial gaps remain in target precision, adaptation to disease heterogeneity, temporal specificity and personalized intervention strategies. Future advances must address these limitations by developing deep-penetrating, adaptive stimulation tools and refining cross-species circuit models to guide individualized treatments.

### Targeted molecular therapies for the glutamate system

Targeted molecular therapies modulating the glutamatergic system are emerging as a promising therapeutic strategy for AD. In addition to the classic NMDA receptor antagonists (such as memantine), other drug treatments targeting glutamate receptors are also being explored [215]. For example, AMPA receptor modulators (such as ampakines) have been shown to enhance synaptic transmission and neuroplasticity, improving cognitive function, particularly spatial memory [216]. Furthermore, positive modulators of NMDA receptor, such as D-cycloserine, a partial agonist of the NMDA receptor, have demonstrated certain therapeutic effects and hold promise in improving learning and memory [217]. Kainate receptor antagonists such as NBQX are also under investigation, aiming to reduce excitotoxicity and improve cognitive function [218]. Additionally, modulators of mGluRs, especially mGluR5 antagonists, have shown potential in regulating synaptic transmission and plasticity [219]. Despite progress in preclinical research, further clinical trials are needed to verify the long-term efficacy and safety of these drugs. Similar to neuromodulation techniques, pharmacological strategies face challenges such as limited target specificity, variability of response across individuals and incomplete understanding of optimal dosing paradigms [220].

Although glutamate receptor modulators and neuromodulation techniques are currently primarily used independently, their combined application may offer new perspectives for the treatment of AD. Glutamate receptor modulators (such as NMDAR antagonists and AMPAR enhancers) can regulate the balance of neurotransmitters, alleviating excessive neural excitability and neurotoxicity, while neuromodulation techniques (such as deep brain stimulation and transcranial magnetic stimulation) promote the recovery of neural circuit function by modulating neural electrical activity. The combination of these two approaches can not only enhance neuroplasticity and synaptic transmission, but also potentially restore the function of the MS–HPC–LS circuit by synchronously modulating the excitability and plasticity of neural circuits, thereby improving cognitive performance. Future research should focus on optimizing the combined application of these methods, exploring their potential for early intervention in AD and providing theoretical support for personalized treatment.

## Conclusion and perspective

In this review, we summarize the anatomical structure of the MS–HPC–LS circuitry, which includes both the MS–HPC and HPC–LS pathways. We discuss the critical roles they play in spatial memory and cognitive functions. Additionally, we describe anatomical alterations of these circuits in AD as well as their impact on spatial memory in mouse models. Furthermore, we elucidate the mechanisms underlying the impairment of these neural circuits. We propose that pathological changes during the progression of AD result in synaptic damage through glutamate receptors, subsequently affecting the integrity of these neural circuits. Abnormal alterations in both the anatomical and functional connectivity of these loops provide a significant neurobiological basis for the spatial memory deficits observed in patients.

Disruption of the MS–HPC connection can occur early in the progression of AD. These abnormalities in circuit connections are reflected not only as structural alterations but also functional imbalances, leading to impaired encoding, storage and retrieval of spatial information in the brain. The HPC–LS neural circuit has been less extensively studied in the context of AD, but emerging evidence suggests that communication between the HPC and the LS is disrupted in the early stages of AD pathology, indicating that damage to this circuit may serve as an early predictor of the disease. Moreover, the MS, HPC and LS are not isolated from each other. The HPC serves as a relay station for MS and LS, and it may connect them to jointly guide spatial memory. Therefore, the integrated function of these three brain regions should not be overlooked. Notably, glutamate receptors in the LS and HPC, which are closely related to cognitive functions, may be integral to the pathological process of AD. Therefore, future studies should focus on how these glutamate receptors regulate the function of the MS–HPC–LS neural circuitry and explore the specific mechanisms by which they contribute to the progression of AD.

In summary, the MS–HPC–LS circuitry is a promising therapeutic target for AD-related spatial memory deficits. Combining neuromodulation techniques such as DBS and TMS with pharmacological treatments targeting glutamate receptors may help restore neural circuit function and improve memory. Future research is needed to optimize these approaches and clarify the molecular mechanisms driving early AD pathology, in order to provide new targets for prevention and early intervention. Beyond current strategies, future research must also address critical technical barriers to advance circuit-targeting neuromodulation. First, neuromodulation approaches such as high-frequency TMS, nTMS and transcranial focused ultrasound require improved depth and spatial precision for modulating deep structures like LS, along with optimized stimulation protocols and imaging guidance [201, 202]. DBS, while promising, remains limited by anatomical variability, surgical risks and lack of standardized parameters, necessitating innovations in electrode design and closed-loop systems. Additionally, the development of AAV-based circuit-specific gene therapies faces challenges including blood–brain barrier permeability and delivery efficiency, which may be addressed by advances in capsid engineering and novel delivery strategies [221]. Finally, identifying translatable biomarkers such as EEG patterns, imaging-based connectivity metrics, or molecular signatures for real-time closed-loop control, remains a key research priority [212]. Overcoming these obstacles may ultimately facilitate early intervention and functional recovery in AD through precise MS–HPC–LS circuitry modulation.

## Data Availability

Not Applicable.

## Abbreviations

MS: Medial septum
HPC: Hippocampus
LS: Lateral septum
AD: Alzheimer's disease
DBB: Diagonal band of Broca
GABA: γ-Aminobutyric acid
GAD: Glutamic acid decarboxylase
PV: Parvalbumin
ChAT: Choline acetyltransferase
SST: Somatostatin
SWRs: Sharp-wave ripples
DG: Dentate gyrus
LTP: Long-term potentiation
dLS: dorsal Lateral Septum
iLS: intermediate Lateral Septum
vLS: ventral Lateral Septum
APP: Amyloid precursor protein
LFP: Local field potential
CNS: Central nervous system
Aβ: β-amyloid
NMDAR: N-methyl-D-aspartate receptor
CP-AMPAR: Calcium-permeable AMPAR
LLPS: Liquid–liquid phase separation
LTD: Long-term depression
PSD-95: Postsynaptic density protein 95
PET: Positron emission tomography
ROS: Reactive oxygen species
mGluR: Metabotropic glutamate receptor
KAR: Kainate receptor
STAT1: Signal transducer and activator of transcription 1
hTau: Human full length tau
DBS: Deep brain stimulation
TMS: Transcranial magnetic stimulation
tDCS: Transcranial direct current stimulation
